# Intestinal Dendritic Cells Specialize to Activate Transforming Growth Factor-β and Induce Foxp3^+^ Regulatory T Cells via Integrin αvβ8

**DOI:** 10.1053/j.gastro.2011.06.057

**Published:** 2011-11

**Authors:** John J. Worthington, Beata I. Czajkowska, Andrew C. Melton, Mark A. Travis

**Affiliations:** ⁎ Manchester Immunology Group and Wellcome Trust Centre for Cell-Matrix Research, Faculty of Life Sciences, University of Manchester, Manchester, United Kingdom; ‡ Lung Biology Center, Department of Medicine, University of California, San Francisco, San Francisco, California

**Keywords:** Immune Response, T-Cell Regulation, Signaling, Inflammatory Response, Self Tolerance, DC, dendritic cell, LAP, latency-associated peptide, mIgG, mouse immunoglobulin G, mLN, mesenteric lymph node, qPCR, quantitative polymerase chain reaction, RA, retinoic acid, TGF, transforming growth factor, Treg, regulatory T cell, iTreg, inducible regulatory T cell

## Abstract

**Background & Aims:**

The intestinal immune system is tightly regulated to prevent responses against the many nonpathogenic antigens in the gut. Transforming growth factor (TGF)-β is a cytokine that maintains intestinal homeostasis, in part by inducing Foxp3^+^ regulatory T cells (Tregs) that suppress immune responses. TGF-β is expressed at high levels in the gastrointestinal tract as a latent complex that must be activated. However, the pathways that control TGF-β activation in the intestine are poorly defined. We investigated the cellular and molecular pathways that control activation of TGF-β and induction of Foxp3^+^ Tregs in the intestines of mice to maintain immune homeostasis.

**Methods:**

Subsets of intestinal dendritic cells (DCs) were examined for their capacity to activate TGF-β and induce Foxp3^+^ Tregs in vitro. Mice were fed oral antigen, and induction of Foxp3^+^ Tregs was measured.

**Results:**

A tolerogenic subset of intestinal DCs that express CD103 were specialized to activate latent TGF-β, and induced Foxp3^+^ Tregs independently of the vitamin A metabolite retinoic acid. The integrin αvβ8, which activates TGF-β, was significantly up-regulated on CD103^+^ intestinal DCs. DCs that lack expression of integrin αvβ8 had reduced ability to activate latent TGF-β and induce Foxp3^+^ Tregs in vitro and in vivo.

**Conclusions:**

CD103^+^ intestinal DCs promote a tolerogenic environment in the intestines of mice via integrin αvβ8-mediated activation of TGF-β.

See editorial on page 1559.

The intestinal immune system encounters a wealth of antigenic stimulation consisting of food substances and commensal bacteria that inhabit the gut.^1^ Regulatory processes must therefore prevent detrimental immune responses to these harmless antigens while still being able to mount protective responses against pathogens that enter the digestive tract. A breakdown in this tight regulation can lead to debilitating autoimmunity and inflammatory bowel disease.

Strong evidence exists that CD4^+^ regulatory T cells (Tregs) play a crucial role in regulating inflammatory responses at environmental interfaces such as the gut.^2^ The most prevalent subset of Tregs, marked by expression of the transcription factor Foxp3, can arise naturally in the thymus during T-cell development (natural Tregs) or can be induced in the periphery from naïve CD4^+^ T cells (inducible Tregs [iTregs]).^3^ Induction of iTregs is dependent on T-cell receptor stimulation and the cytokine transforming growth factor (TGF)-β^4^ and has been proposed to be important in maintaining gut immune tolerance.^2^ However, the mechanisms underlying iTreg induction in the gut are poorly understood.

Given their fundamental importance in regulation of T-cell responses, dendritic cells (DCs) have been suggested to play a central role in regulating Foxp3^+^ Treg responses and tolerance in the intestine.^5^ Acting as the sentinels of the gut, these cells are decisively positioned throughout the intestine to capture luminal contents and process and present these antigens to T cells within the gut-draining mesenteric lymph node (mLN). Several lines of investigation have indicated that DCs marked by the expression of CD103 have important tolerogenic properties, with reduced production of proinflammatory cytokines and an enhanced ability to induce Foxp3^+^ iTregs.^6,7^ The ability of CD103^+^ intestinal DCs to induce iTregs has been linked to their ability to produce enhanced levels of the dietary metabolite retinoic acid (RA) via enhanced expression of retinal dehydrogenase *aldh1a2*.^6,7^ Such RA-mediated iTreg induction by CD103^+^ intestinal DCs requires synergy with the key immunoregulatory cytokine TGF-β. TGF-β is highly expressed in the intestine but importantly is always produced as a latent protein complex that must be activated to exert biologic function.^8^ However, the cellular and molecular mechanisms that regulate TGF-β activity and iTreg induction in the intestine are not known.

In this study, we show that intestinal CD103^+^ DCs are specialized to generate Foxp3^+^ iTregs independent of the actions of RA. We found that CD103^+^ DCs from the intestine have an increased ability to activate latent TGF-β that is directly responsible for their increased ability to induce iTregs. Furthermore, we find that intestinal CD103^+^ DCs express greatly elevated levels of the TGF-β–activating integrin αvβ8, which is absolutely required for both their enhanced ability to activate latent TGF-β and their specialized ability to induce iTregs in vitro and in vivo. These results highlight a novel mechanism by which CD103^+^ DCs in the intestine promote Foxp3^+^ Treg induction and bring to the forefront integrin-mediated TGF-β activation in promoting tolerance within the gut.

## Materials and Methods

### Animals

Mice lacking integrin αvβ8 on DCs via expression of a conditional floxed allele of β8 integrin in combination with CD11c-Cre (*Itgb8 (CD11c-Cre)* mice) have been previously described.^9^ OT-II/Rag^−/−^ and Foxp3^GFP^ mice^10^ were kind gifts from Dr K Okkenhaug (Babraham Institute, Cambridge, England) and Dr A. Rudensky (Memorial Sloan-Kettering Cancer Center, New York, NY), respectively. All mice were maintained in specific pathogen-free conditions at the University of Manchester and used at 6 to 8 weeks of age. All experiments were performed under the regulations of the Home Office Scientific Procedures Act (1986).

### Purification of DCs

Mouse mLN or spleen was incubated with shaking for 20 minutes at 37°C in RPMI-1640 with 0.08 U/mL Liberase Blendzyme 3 (Roche, Burgess Hill, United Kingdom) or 1 mg/mL collagenase VIII and 50 U/mL deoxyribonuclease I, respectively. Small/large intestinal lamina propria were excised and prepared as described.^11^ Cells were blocked with anti-FcγR antibody (clone 24G2) before enrichment using a CD11c enrichment kit (Miltenyi Biotec, Bisley, United Kingdom). To purify CD103^+/−^ DCs, enriched DCs were labeled with anti-CD103 (M290) and anti-CD11c (N418) antibodies and sorted using a FACSAria (BD Biosciences, San Jose, CA). In all experiments, subset purity was >95%.

### T-Cell Purification

Splenocytes from Foxp3^GFP^ mice were stained with anti-CD4 (GK1.5) and anti-CD44 (IM7) antibodies and CD4^+^ CD44^−^/low, GFP^−^ populations sorted using a FACSAria. Cell purity in all experiments was >99.8%.

### In Vitro Treg Induction Assay

A total of 5 × 10^4^ CD4^+^ CD44^−^/low, Foxp3^GFP−^ T cells isolated from Foxp3^GFP^ mice were cultured with 2.5 × 10^3^ CD103^+/−^ DC subsets in RPMI 1640 media (+10% FBS, 1% penicillin/streptomycin, 1% l-glutamine, 50 μm 2-mercaptoethanol) with 0.06 μg/mL α-CD3 antibody for 5 days with addition of 5 ng/mL recombinant human interleukin-2 every other day. Induction of CD4^+^ Foxp3^GFP+^ Tregs was analyzed by flow cytometry, with cells stained with anti-CD4 and α4β7 (DATK-32) antibodies. Cell viability was assessed using 7-AAD. In addition, 40 μg/mL control mouse immunoglobulin G (mIgG) or α–TGF-β antibody (clone 1D11), 2 ng/mL recombinant human TGF-β, 100 nmol/L all-trans RA, and/or 1 μmol/L RA receptor inhibitors LE540 and LE135 were added as indicated.

### In Vivo Treg Assay

CD4^+^ T cells from OTII/Rag^−/−^ mouse spleens were enriched using a CD4^+^ enrichment kit and AutoMACS (Miltenyi Biotec), stained with anti-CD4 and Vα2 (B20.1) antibodies, and sorted for CD4^+^, Vα2^+^ cells on a FACSAria. Purity obtained was >99.8% in all experiments. Cells were labeled with 2 μmol/L carboxyfluorescein succinimidyl ester, 2 × 10^6^ cells injected intravenously into control or *Itgb8* (*CD11c*-Cre) recipient mice, and mice fed ovalbumin (10 mg/mL) in drinking water for 5 days. On day 6, spleen/lymph node cells were harvested and stained with anti-CD4, Vα2, and Foxp3 (FJK-16s) antibodies. Induced carboxyfluorescein succinimidyl ester–labeled Foxp3^+^ cells were detected by flow cytometry.

### TGF-β Activation Assay

CD103^+/−^ DCs were incubated with mink lung epithelial cells transfected with a plasmid containing firefly luciferase complementary DNA downstream of a TGF-β–sensitive promoter^12^ in the presence of 1 μg/mL lipopolysaccharide. Cocultures were incubated overnight in the presence of 40 μg/mL control mIgG or anti–TGF-β antibody (clone 1d11) and luciferase detected via the Luciferase Assay System (Promega, Southampton, United Kingdom). TGF-β activity was determined as the difference in luciferase activity between control mIgG-treated samples and samples treated with anti–TGF-β antibody.

### Quantitative Polymerase Chain Reaction

Total RNA was purified from sorted DC subsets using an RNeasy Mini Kit (Qiagen, Crawley, United Kingdom). RNA was reverse transcribed using oligo(dT) primers and complementary DNA for specific genes detected using a SYBR Green qPCR Kit (Finnzymes, Vantaa, Finland). Gene expression was normalized to HPRT levels (see Supplementary Table 1 for primers used).

### Statistical Analysis

Results are expressed as mean ± SEM. Where statistics are quoted, 2 experimental groups were compared using the Student *t* test for nonparametric data. Three or more groups were compared using the Kruskal–Wallis test, with Dunn's multiple comparison posttest. *P* ≤ .05 was considered statistically significant.

## Results

### The Enhanced Ability of Intestinal CD103^+^ DCs to Induce Foxp3^+^ iTregs Is Due to an Increased Ability to Activate TGF-β and Does Not Require RA

**Figure 1:**
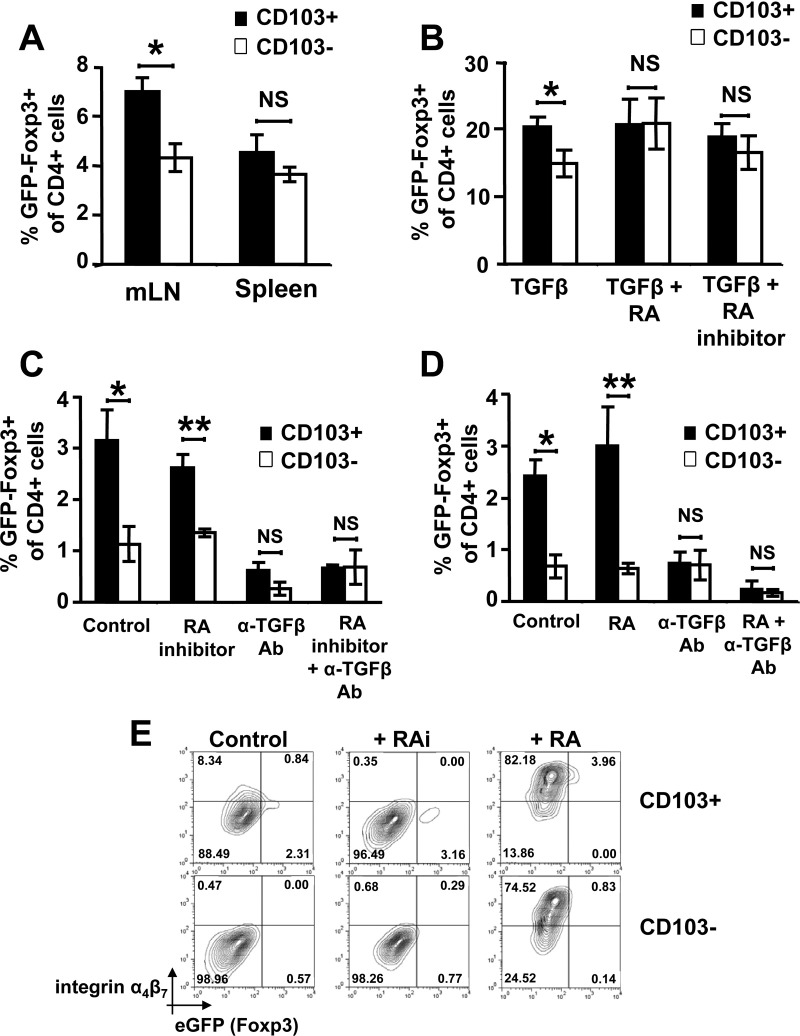
RA is not essential for the enhanced ability of CD103^+^ intestinal DCs to induce Tregs. (*A*–*E*) A total of 5 × 10^4^ CD4^+^ Foxp3^GFP−^ T cells were incubated with 2.5 × 10^3^ CD103^+/−^ DCs in the presence of anti-CD3 antibody and/or RA, RA inhibitors, and/or anti–TGF-β antibody, and the induction of iTregs (CD4^+^ Foxp3^GFP+^) was assessed by flow cytometry after 5 days. (*A*) CD103^+/−^ DC subsets from mLN or spleen in the absence of exogenous TGF-β. (*B*) CD103^+/−^ DCs from mLN in the presence of 2 ng/mL TGF-β and 100 nmol/L RA or 1 μmol/L of RA inhibitors (LE-135, LE-450). (*C*) CD103^+/−^ DCs from mLN in the absence of exogenous active TGF-β and/or 1 μmol/L RA inhibitors and/or 40 μg/mL anti–TGF-β antibody. (*D*) CD103^+/−^ DCs from mLN in the presence of 100 nmol/L RA and/or 40 μg/mL anti–TGF-β antibody. Data are representative of at least 3 independent experiments. *P < .05, **P < .01. (*E*) Representative flow cytometry plots from *C* and *D* analyzing effects of RA inhibitors and RA on CD4^+^ T-cell expression of Foxp3^GFP^ and α_4_β_7_.

Recent data have indicated that a CD103^+^ subset of intestinal DCs promotes de novo generation of Foxp3^+^ iTregs.^6,7^ However, the molecular mechanisms driving this process are not clear. We first addressed whether enhanced induction of Foxp3^+^ iTregs was a general property of CD103^+^ DCs or whether this property was distinct to intestinal DCs. As previously shown,^6,7^ we found that CD103^+^ DCs isolated from the gut-draining mLN have an enhanced ability to induce Foxp3^+^ iTregs compared with CD103^−^ DCs (Figure 1*A*). However, CD103^+/−^ subsets isolated from spleen showed an equivalent capacity to induce Foxp3^+^ iTregs, similar to levels seen with CD103^−^ DCs from mLN (Figure 1*A*). Hence, enhanced Foxp3^+^ iTreg induction seems to be a specific property of intestinal CD103^+^ DCs rather than a general property of CD103^+^ DCs.

Because CD103^+^ DCs from the intestine are specialized to induce Foxp3^+^ iTregs, we sought to identify novel mechanisms responsible for this property. Work has proposed that the enhanced ability of CD103^+^ intestinal DCs to induce iTregs is due to the elevated production of the vitamin A metabolite RA, resulting from elevated expression of the RA-metabolizing enzyme ALDH1a2.^7^ Indeed, as previously reported,^6,7^ in the presence of exogenous active TGF-β, the enhanced ability of intestinal CD103^+^ DCs to induce Foxp3^+^ iTregs is prevented by the inhibition of RA (Figure 1*B*). Additionally, RA rescues the ability of CD103^−^ DCs to induce iTregs to levels seen with CD103^+^ DCs (Figure 1*B*). However, because these and previous experiments involve addition of high levels of exogenous active TGF-β, the relative contribution of TGF-β/RA to enhanced induction of iTregs by intestinal CD103^+^ DCs cannot be assessed. To address this point, we repeated experiments in the absence of exogenous active TGF-β. Interestingly, when RA was inhibited in the absence of exogenous TGF-β, CD103^+^ intestinal DCs were still capable of inducing elevated levels of Foxp3^+^ iTregs versus CD103^−^ intestinal DCs (Figure 1*C*). Similarly, addition of exogenous RA to cultures did not rescue the ability of CD103^−^ DCs to induce iTregs to levels seen with CD103^+^ DCs (Figure 1*D*). This was despite the fact that the induction of RA-dependent gut-homing receptor α_4_β_7_ was blocked by RA inhibition and induced by addition of RA (Figure 1*E*). Strikingly, however, antibody-mediated blockade of TGF-β function completely abrogated the enhanced ability of intestinal CD103^+^ DCs to induce Foxp3^+^ Tregs under all conditions (Figure 1*C* and *D*). Collectively, these data show for the first time that intestinal CD103^+^ DCs can preferentially induce Foxp3^+^ iTregs independently of RA and this enhanced induction is totally dependent on TGF-β. We therefore sought to identify novel mechanisms responsible for the specialization of intestinal CD103^+^ DCs to generate iTregs.

**Figure 2:**
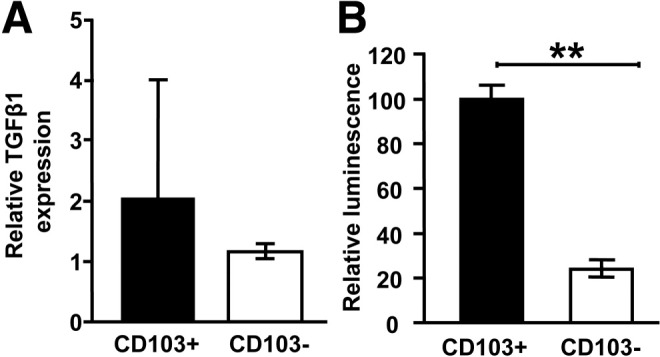
CD103^+^ intestinal DCs have an increased ability to activate latent TGF-β. (*A*) Latent TGF-β production by CD103^+/−^ mLN DCs was assessed by qPCR and levels normalized to the housekeeping gene HPRT. Data represent 3 independent experiments. (*B*) Production of active TGF-β by CD103^+/−^ DCs from mLN was assessed by culturing DCs with an active TGF-β reporter cell line.^12^ Data represent 3 independent experiments. **P < .01.

Because TGF-β is produced as a latent complex that must be activated to function, we hypothesized that intestinal CD103^+^ DCs either produce elevated levels of latent TGF-β or are specialized to activate TGF-β. To test these hypotheses, we directly measured the ability of intestinal CD103^+/−^ DCs to produce and activate latent TGF-β. Using quantitative polymerase chain reaction (qPCR), we found no significant increase in the ability of CD103^+^ intestinal DCs to produce latent TGF-β messenger RNA versus CD103^−^ intestinal DCs (Figure 2*A*). Importantly, however, using a bioassay to detect the activated form of TGF-β,^12^ intestinal CD103^+^ DCs showed a greatly enhanced ability to activate latent TGF-β when compared with CD103^−^ DCs (Figure 2*B*). These results strongly suggest that elevated Foxp3^+^ iTreg induction by intestinal CD103^+^ DCs is driven by their enhanced ability to activate latent TGF-β.

### Elevated Expression of the Integrin αvβ8 by Intestinal CD103^+^ DCs Is Responsible for Their Enhanced Ability to Activate TGF-β

**Figure 3:**
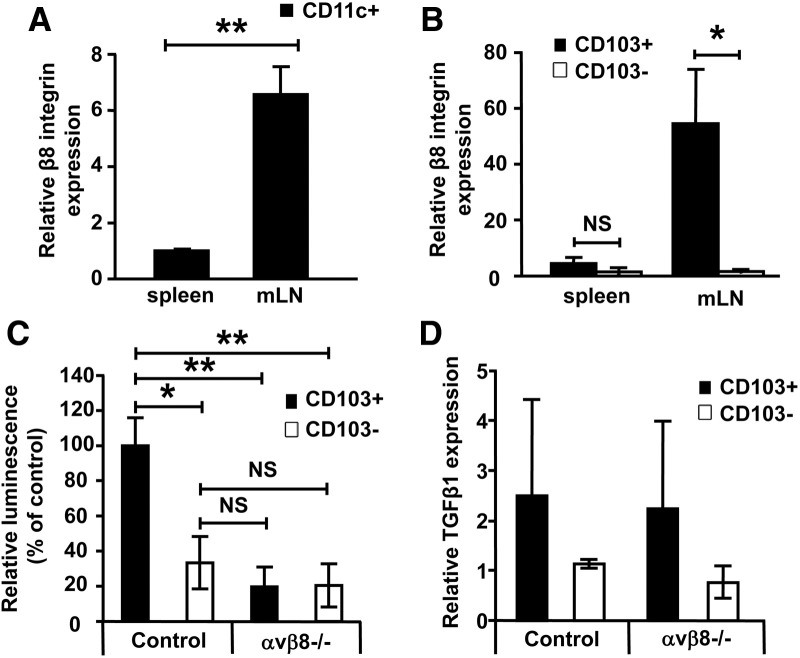
CD103^+^ intestinal DCs express elevated levels of the TGFβ-activating integrin αvβ8 and activate more TGF-β in an integrin αvβ8-dependent manner. RNA from (*A*) total DCs or (*B*) splenic/mLN CD103^+/−^ DCs was analyzed for integrin β8 expression by qPCR. β8 levels were normalized to the housekeeping gene HPRT and presented relative to levels in total spleen DCs (for *A*) or spleen CD103-ve DCs (for *B*). Data shown are representative of 2 and 4 independent experiments, respectively. (*C*) TGF-β activation by control or αvβ8^−/−^ CD103^+/−^ mLN DCs was detected by coculture with an active TGF-β reporter cell line.^12^ Data represent 5 independent experiments. (*D*) RNA from control or αvβ8^−/−^ CD103^+/−^ DCs was analyzed for TGF-β expression by qPCR. Data shown represent 2 independent experiments. *P < .05, **P < .01.

We next aimed to determine the mechanisms that support enhanced latent TGF-β activation by intestinal CD103^+^ DCs. Recent evidence has highlighted an important role for specific integrin receptors in modulating activation of TGF-β via binding to an RGD integrin binding motif present in the latency-associated peptide (LAP) region of latent TGF-β.^13^ When we analyzed total CD11c^+^ DCs, we saw a marked increase in expression of the TGF-β–activating integrin receptor αvβ8 on DCs isolated from mLN compared with spleen (Figure 3*A*). Strikingly, we found a highly significant (∼50-fold) increase in expression levels of integrin αvβ8 on intestinal CD103^+^ DCs compared with CD103^−^ DCs (Figure 3*B*). Enhanced expression of integrin αvβ8 appeared specific to intestinal CD103^+^ DCs, because splenic CD103^+/−^ DC subsets showed equivalent expression of integrin αvβ8, similar to levels seen in intestinal CD103^−^ DCs (Figure 3*B*).

To test the functional role of increased integrin αvβ8 expression by intestinal CD103^+^ DCs, we utilized DC subsets isolated from *Itgb8* (*CD11c*-Cre) conditional KO mice that specifically lack integrin αvβ8 on CD11c^+^ DCs.^9^ We found that the enhanced ability of intestinal CD103^+^ DCs to activate latent TGF-β was completely ablated in αvβ8^−/−^ CD103^+^ DCs (Figure 3*C*). Indeed, the level of TGF-β activation seen by αvβ8^−/−^ intestinal CD103^+^ DCs was similar to that seen with wild-type CD103^−^ DCs (Figure 3*C*). Importantly, such reduced TGF-β activation was not due to a decreased ability to produce latent TGF-β, because expression of latent TGF-β by control and αvβ8-deficient DCs was similar (Figure 3*D*). Therefore, enhanced expression of integrin αvβ8 by intestinal CD103^+^ DCs is critical for the increased ability of these cells to activate latent TGF-β.

### The Enhanced Ability of Intestinal CD103^+^ DCs to Induce Foxp3^+^ iTregs Is Wholly Dependent on TGF-β Activation by Integrin αvβ8

**Figure 4:**
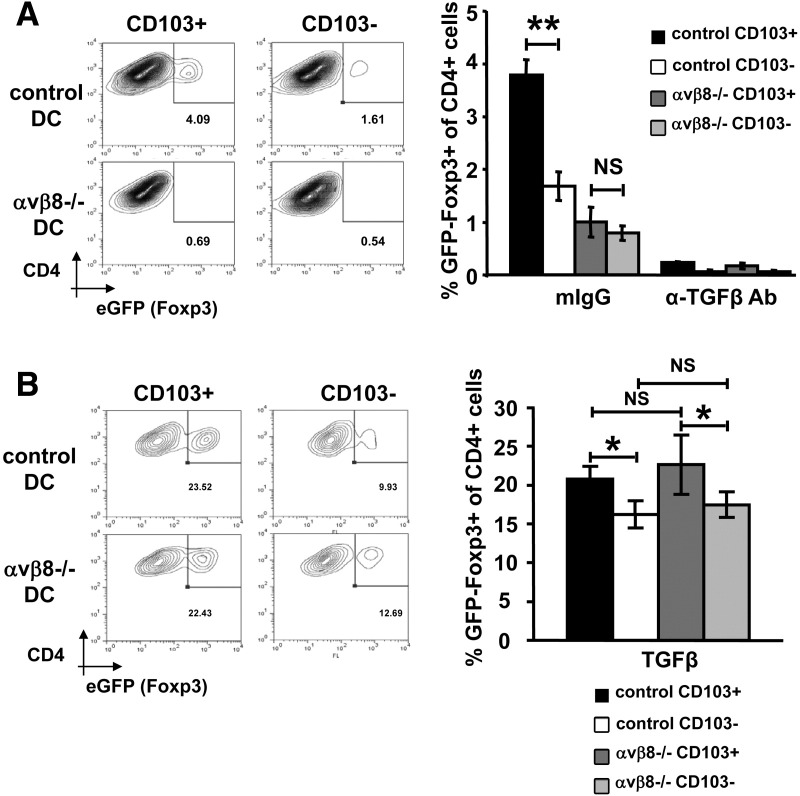
Integrin αvβ8-mediated TGF-β activation is critical for the enhanced ability of CD103^+^ intestinal DCs to induce Foxp3^+^ Tregs. A total of 5 × 10^4^ CD4^+^ Foxp3^GFP−^ T cells were cultured with 2.5 × 10^3^ control or αvβ8^−/−^ CD103^+/−^ DC subsets in the presence of anti-CD3 antibody plus (*A*) 40 μg/mL mIgG or anti–TGF-β antibody or (*B*) 2 ng/mL exogenous active TGF-β. Foxp3^+^ Treg induction was assessed by flow cytometry after 5 days. Representative flow cytometry plots and data are shown from 3 or 5 independent experiments, respectively. *P < .05, **P < .01.

To assess if increased expression of the TGF-β–activating αvβ8 integrin on intestinal CD103^+^ DCs was responsible for their enhanced ability to induce Foxp3^+^ iTregs, we compared the ability of αvβ8^−/−^ intestinal DC subsets to induce iTregs ex vivo. In the absence of integrin αvβ8, the enhanced ability of intestinal CD103^+^ DCs to induce Foxp3^+^ iTregs was completely ablated, similar to levels seen for CD103^−^ DCs (Figure 4*A*). Importantly, the addition of exogenous active TGF-β completely rescued iTreg induction by αvβ8^−/−^ intestinal CD103^+^ DCs to levels seen with control CD103^+^ DC subsets (Figure 4*B*). Addition/inhibition of RA failed to rescue the ability of αvβ8^−/−^ intestinal CD103^+^ DCs to induce iTregs (Supplementary Figure 1A and B). Indeed, the absence of αvβ8 integrin did not affect the increased expression of aldh1a2 messenger RNA by intestinal CD103^+^ DCs or their increased ability to induce the gut homing receptor α_4_β_7_ on responding CD4^+^ T cells (Supplementary Figure 2A and B), showing that lack of integrin αvβ8 does not affect the enhanced ability of CD103^+^ intestinal DCs to produce RA. αvβ8^−/−^ CD103^+^ DCs also showed reduced production of inflammatory cytokines compared with CD103^−^ DCs^6^ (Supplementary Figure 3), indicating that reduced TGF-β activation by αvβ8 does not result in an overt proinflammatory phenotype in CD103^+^ intestinal DCs.

**Figure 5:**
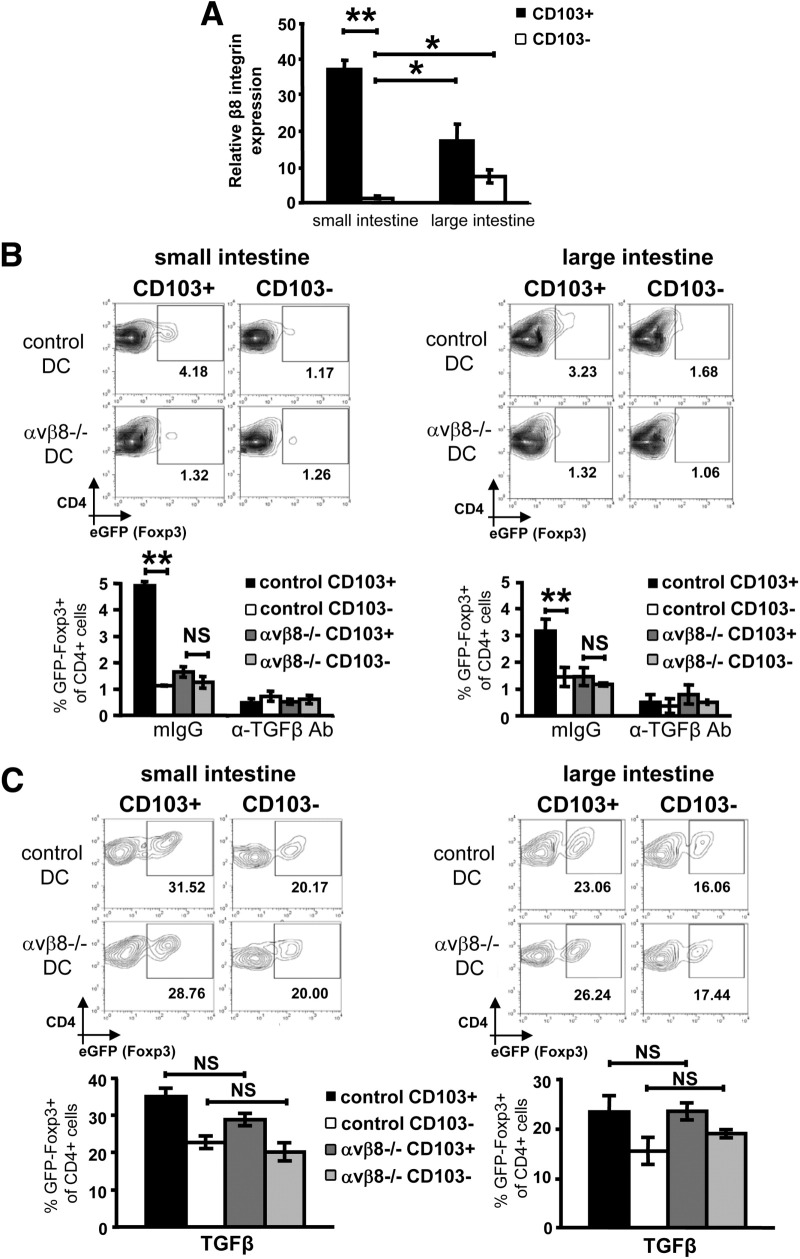
Elevated expression of integrin αvβ8 by small and large intestinal lamina propria CD103^+^ DCs is required for enhanced induction of Foxp3^+^ Tregs. (*A*) Integrin β8 expression in small/large intestinal lamina propria CD103^+/−^ DCs was assessed by qPCR, normalizing levels to the housekeeping gene HPRT. Data are presented as relative to small intestinal CD103-ve DC levels and represent 3 independent experiments. (*B* and *C*) A total of 5 × 10^4^ CD4^+^ Foxp3^GFP−^ T cells were cultured with 2.5 × 10^3^ control or αvβ8^−/−^ CD103^+/−^ DC subsets in the presence of anti-CD3 antibody plus (*B*) mIgG or anti–TGF-β antibody or (*C*) exogenous active TGF-β. Foxp3^+^ Treg induction was assessed by flow cytometry after 5 days. Representative flow cytometry plots and data are shown from 2 to 6 independent experiments. *P < .05, **P < .01.

Data presented previously were obtained using intestinal DC subsets isolated from mLN, which include DCs draining from the small and large intestine. To determine whether CD103^+^ DCs present within intestinal tissues show a similar reliance on integrin αvβ8-mediated TGF-β activation to induce Foxp3^+^ iTregs, we first analyzed expression of β8 integrin on DCs isolated from small and large intestinal lamina propria. Similar to mLN DC subsets, CD103^+^ DCs from both the small and large intestine expressed high levels of β8 integrin (Figure 5*A*). Additionally, CD103^+^ DCs from both small and large intestine supported enhanced Foxp3^+^ iTreg induction versus CD103^−^ DCs, which was completely reliant on expression of integrin αvβ8 (Figure 5*B*). As observed for mLN, iTreg induction in αvβ8^−/−^ CD103^+^ DCs from small or large intestine was rescued by addition of active TGF-β (Figure 5*C*). Interestingly, we observed slightly elevated expression of β8 on CD103^−^ DCs from large intestinal lamina propria versus CD103^−^ DCs from small intestine (Figure 5*A*), mLN, and spleen (data not shown). However, such expression did not translate into an enhanced ability to induce iTreg, indicating a potentially novel role for β8 expression on CD103^−^ DCs from the large intestine (Figure 5*B*). Taken together, these data show that increased αvβ8-mediated TGF-β activation by intestinal CD103^+^ DCs is critical for their enhanced ability to induce Foxp3^+^ iTregs ex vivo.

### The Absence of Integrin αvβ8 on Intestinal DCs Leads to an Absence of Foxp3^+^ iTreg Induction In Vivo During Oral Administration of Antigen

We next sought to determine whether integrin αvβ8 expression by intestinal DCs supported enhanced Foxp3^+^ iTreg induction in vivo. To this end, we adoptively transferred ovalbumin antigen-specific CD4^+^ OT-II T cells into control or *Itgb8* (*CD11c*-Cre) mice and supplemented drinking water with ovalbumin. T cells were isolated from OT-II/Rag^−/−^ mice, which lack endogenous Foxp3^+^ Tregs. Previous experiments using this method have shown that Foxp3^+^ iTreg induction is promoted specifically in the mLN, at least in part via the enhanced ability of intestinal CD103^+^ DCs to promote iTreg induction.^6,7^

**Figure 6:**
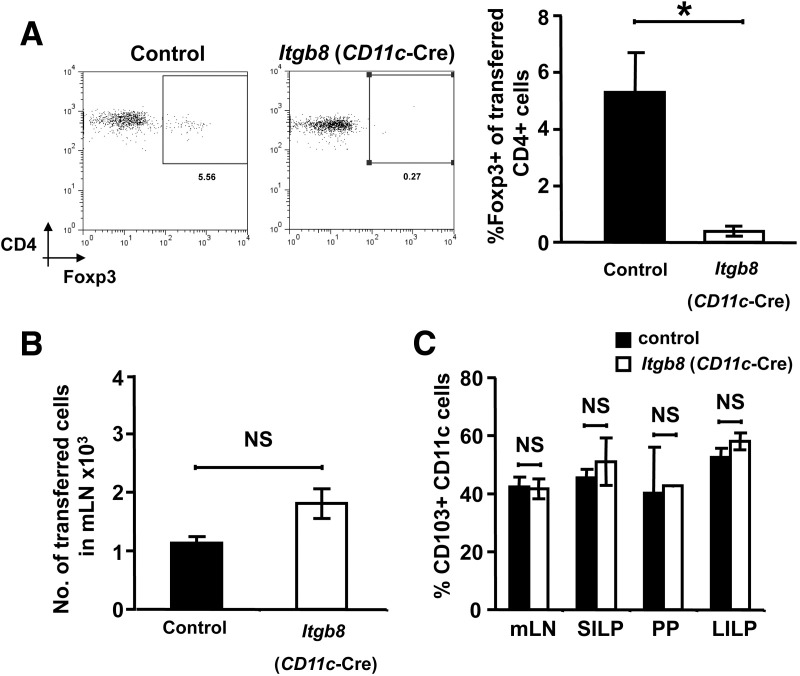
Integrin αvβ8-mediated TGF-β activation is critical for the enhanced ability of intestinal DCs to induce Foxp3^+^ Tregs in vivo after oral administration of antigen. A total of 2 × 10^6^ carboxyfluorescein succinimidyl ester–labeled CD4^+^ T cells from OTII/Rag^−/−^ mice were injected intravenously into control or *Itgb8* (*CD11c-Cre*) mice. Recipient mice were fed ovalbumin in drinking water and mLN analyzed for CD4^+^ Vα2^+^ Foxp3^+^ cells after 6 days. (*A*) Representative flow cytometry plots and pooled data showing iTreg in mLN of recipient mice (n = 3). (*B*) Total number of transferred cells in mLN of recipient mice. (*C*) Proportion of CD103^+^ DCs in the mLN, small intestine lamina propria (SILP), Peyer's patches (PP), and large intestine lamina propria (LILP) of control and *Itgb8* (*CD11c-Cre*) mice. Data from 8 independent experiments. *P < .05.

In control mice, we observed ∼5% induction of Foxp3^+^ iTregs arising from adoptively transferred OT-II T cells specifically within the mLN (Figure 6*A*). This induced population was not observed in the spleen or in mice not fed ovalbumin (data not shown). Strikingly, induction of Foxp3^+^ iTregs in mLN was completely absent in mice lacking integrin αvβ8 on DCs (Figure 6*A*), despite a similar number of transferred CD4^+^ T cells being detected in the mLN of *Itgb8* (*CD11c*-Cre) and control mice (Figure 6*B*). Because TGF-β can induce expression of CD103 in some cells,^14^ a potential explanation for the reduced ability of *Itgb8* (*CD11c*-Cre) mice to induce iTregs is that lower CD103^+^ DC numbers are present in these mice owing to reduced TGF-β activation. However, we found that *Itgb8* (*CD11c*-Cre) mice had comparable numbers of CD103^+^ DCs in all gut-associated lymphoid tissue examined (Figure 6*C*). Taken together with our in vitro data, these results strongly indicate that αvβ8-mediated TGF-β activation by specialized intestinal CD103^+^ DCs is essential for the induction of tolerogenic Foxp3^+^ iTregs in the gut.

## Discussion

Intestinal CD103^+^ DCs have emerged as key cells in maintaining gut tolerance, with recent data showing that these cells have the enhanced ability to induce gut-homing receptors on responding T cells^15^ and convert naïve T cells to immune-suppressive Foxp3^+^ iTregs.^6,7^ These important functions appear to be due to high expression of the retinal dehydrogenase *aldh1a2* in CD103^+^ intestinal DCs, suggesting they have the capacity to metabolize retinal acid to RA.^6^ However, our data now show that CD103^+^ gut DCs have an enhanced ability to induce iTregs that is independent of RA but completely dependent on TGF-β function. These results strongly suggest that the enhanced ability of CD103^+^ intestinal DCs to induce iTregs is linked to an increased ability of these cells to produce active TGF-β. Indeed, we directly show for the first time that CD103^+^ intestinal DCs are specialized to activate latent TGF-β and that elevated expression of the TGF-β–activating integrin αvβ8 by CD103^+^ intestinal DCs is responsible for the enhanced ability of these cells to activate latent TGF-β. Importantly, elevated integrin αvβ8-mediated TGF-β activation by CD103^+^ intestinal DCs is responsible for their increased ability to induce Foxp3^+^ Tregs both in vitro and in vivo. We have therefore identified a novel molecular pathway by which a specialized gut DC subset activates TGF-β to promote a tolerogenic environment via induction of Foxp3^+^ iTregs.

Many different immune cells produce TGF-β (predominately the isoform TGF-β1^16^) but always noncovalently bound to an N-terminal propeptide (LAP), preventing TGF-β binding to its receptor.^8^ Hence, TGF-β function is exquisitely regulated at the level of TGF-β activation. Strong evidence in vivo now supports a critical role for integrin receptors in activating latent TGF-β1 via interaction with an RGD integrin binding motif present in the LAP region of the latent complex.^17^ Our finding that the TGF-β–activating integrin αvβ8 is highly expressed and functionally important on specialized tolerogenic DCs in the intestine correlates with our previous findings that *Itgb8* (*CD11c*-Cre) mice develop severe colitis associated with reduced levels of total Foxp3^+^ Tregs in the colonic lamina propria.^9^ Indeed, this reduction is likely due to an absence of iTregs as opposed to natural Tregs, because we now show that *Itgb8* (*CD11c*-Cre) mice fail to establish an iTreg population in the mLN upon antigen stimulation of adoptively transferred CD4^+^ T cells. Our results therefore indicate that elevated expression of integrin αvβ8 by CD103^+^ intestinal DCs plays an important role in preventing gut inflammation via induction of Foxp3^+^ iTregs.

In addition to activation by integrins, several other mechanisms of TGF-β activation have been proposed, including cleavage by the protease plasmin, MMP2 and MMP9, and interaction with thrombospondin-1.^8^ However, mice lacking these molecules show mild/no inflammation of the gut, indicating a minimal role in the activation of TGF-β to maintain intestinal homeostasis.^18–20^ A previous study has proposed that enhanced production of the TGF-β isoform TGF-β2, latent associated binding protein 3 (LTBP3), and tissue plasminogen activator (tPA) by CD103^+^ intestinal DCs may play roles in enhanced Foxp3^+^ iTreg induction.^6^ However, TGF-β2 does not contain the RGD integrin binding motif that would allow engagement with integrin αvβ8 and Ltbp-3 and tPA^−^/^−^ mice do not develop signs of colitis akin to mice lacking αvβ8 on DCs.^9,21^ Therefore although CD103^+^ intestinal DCs express an abundance of factors involved in TGF-β availability, our data clearly show that αvβ8-mediated TGF-β activation is the critical activator of TGF-β responsible for enhanced Treg induction in the intestine. Interestingly, in lung cancer cells, it has been proposed that activation of TGF-β by integrin αvβ8 involves presentation of the latent complex to the membrane metalloprotease MT1-MMP.^22^ However, we find no evidence for increased expression of MT1-MMP in CD103^+^ intestinal DCs (Supplementary Figure 4). Hence, how CD103^+^ DC-expressed integrin αvβ8 activates latent TGF-β requires further investigation.

An important unanswered question is what is the key cellular source of the TGF-β that is activated by integrin αvβ8-expressing CD103^+^ intestinal DCs? CD103^+^ intestinal DCs show enhanced Foxp3^+^ iTreg induction when cultured with purified CD4^+^ T cells, indicating that TGF-β production by either (or both) of these cell types is sufficient to support iTreg induction. Interestingly, in mice lacking TGF-β expression specifically in T cells, total intestinal Foxp3^+^ Treg numbers were unaltered, suggesting that a TGF-β source other than T cells may be important in maintaining and/or inducing Foxp3^+^ Tregs in the gut.^23^ However, despite similar Foxp3^+^ Treg numbers, in the absence of T cell–derived TGF-β, Foxp3 expression levels in Tregs from the colonic lamina propria were decreased, indicating that T cell–derived TGF-β may play some role in promoting Foxp3^+^ Tregs in the gut.^23^ Alternatively, as shown in Figure 2, we found that CD103^+^ intestinal DCs were able to activate more latent TGF-β than the CD103^−^ subset when DCs were cultured only in the presence of a TGF-β reporter cell line, indicating that DC-expressed TGF-β may be sufficient for the specialized ability of these cells to activate TGF-β.

In agreement with other studies,^7,24,25^ our data suggest that RA is not sufficient to cause enhanced Foxp3^+^ iTreg induction by CD103^+^ intestinal DCs, and we now show that RA can be dispensable for this function. Because enhanced iTreg induction by intestinal CD103^+^ DCs is wholly dependent on their enhanced ability to activate TGF-β, an important question therefore is what are the physiologic situations when RA can act to enhance iTreg conversion in vivo? Studies have shown that RA acts through the RARα receptor expressed on T cells to enhance TGF-β–mediated Foxp3 induction^26–29^ but that mice lacking RARα show normal Foxp3^+^ Treg levels in the lamina propria.^27^ Also, mice fed a vitamin A–deficient diet from birth do not show reduced Foxp3^+^ Treg numbers in the gut, at least in the small intestine.^30^ These data suggest that the role of RA in regulating steady-state levels of Foxp3^+^ Tregs in the gut is minimal. This is in contrast to the role of integrin αvβ8-mediated TGF-β activation, because mice lacking this TGF-β–activating integrin on DCs not only show reduced levels of lamina propria Foxp3^+^ Tregs, but also develop severe colitis under steady-state conditions.^9^

It is conceivable that RA acts to enhance Foxp3^+^ iTreg induction by CD103^+^ intestinal DCs when TGF-β levels are up-regulated (eg, during the course of infection and inflammation).^31^ An important function of RA is its ability to inhibit TGF-β–driven induction of proinflammatory IL-17–producing Th17 cells.^25^ Interestingly, our recent data and that of others have highlighted an important role for integrin αvβ8-mediated TGF-β activation by DCs in promoting Th17 cell induction in mice.^32,33^ Hence, RA may act as an important regulator of Th17-mediated pathology in the gut, acting to dampen integrin αvβ8-mediated TGF-β activation–driven Th17 cell induction by CD103^+^ intestinal DCs during inflammatory responses.

It has been proposed that RA can enhance Foxp3^+^ iTreg induction indirectly by suppressing inflammatory cytokine production by CD4^+^ CD44^hi^ memory T cells.^27^ These data would again support a role for RA in enhancing iTreg induction during active immune responses, via inhibition of inflammatory cytokine production by effector/memory T cells.^27^ However, all iTreg induction experiments described here were performed with naive CD4^+^, CD44^−^/low, Foxp3^−^ T cells, with enhanced iTregs still induced by CD103^+^ intestinal DCs in the absence/presence of RA. We have also performed similar assays, including CD44^hi^ T cells in culture, and again alterations in RA function did not alter the enhanced iTreg induction by CD103^+^ intestinal DCs (Supplementary Figure 5 and data not shown). Our data therefore support a model where RA acts to enhance TGF-β–mediated iTreg induction during inflammatory events, either by directly enhancing Foxp3 transcription,^28^ blocking inhibitory transcriptional signals that prevent Foxp3 production,^25,34^ enhancing TGF-β signaling,^35–37^ or a combination of these.^35^ More work is therefore required to determine the in vivo situations where RA acts to enhance Treg induction in the gut.

It is interesting to postulate which factors condition CD103^+^ intestinal DCs to express elevated integrin αvβ8 levels and why CD103^−^ intestinal DCs avoid similar conditioning. CD103 binds to E-cadherin on intestinal epithelial cells, which will expose CD103^+^ DCs to an array of cytokines that epithelial cells constitutively express during homeostatic conditions. Such factors include thymic stromal lymphopoietin, interleukin-10, RA, and TGF-β itself,^38^ which alter DC function and could potentially up-regulate integrin αvβ8 expression. Similarly, activation of TLR ligands by the microflora could enhance αvβ8 expression by DCs. It is probable that both CD103^+^ and CD103^−^ intestinal DC subsets respond to similar conditions in different ways as they arise from different hard-wired precursors.^15^ Interestingly, CD103^−^ DCs from large intestinal lamina propria showed a slight elevation in β8 expression compared with CD103^−^ DCs from small intestine (Figure 5*A*), mLN, and spleen (data not shown), but this enhanced integrin β8 expression did not result in enhanced iTreg induction. These findings suggest a different functional role for β8 expression (and subsequent TGF-β activation) in these cells, which we are currently investigating.

In conclusion, we have identified for the first time that CD103^+^ intestinal DCs express increased levels of the integrin αvβ8, which is directly responsible for an increased activation of TGF-β, leading to an increased ability to induce Foxp3^+^ iTregs in the steady state that is independent of RA. Our data highlight a novel mechanism in maintaining intestinal homeostasis and offer potential specific treatments to modulate TGF-β function, via the manipulation of αvβ8 integrin, to influence Treg numbers during inflammatory diseases of the intestine.
