# Supplementary material for: Intestinal Dendritic Cells Specialize to Activate Transforming Growth Factor-β and Induce Foxp3+ Regulatory T Cells via Integrin αvβ8
Source: Gastroenterology. 2011 Nov;141(5):1802–12. doi: 10.1053/j.gastro.2011.06.057 (PMC3507624; doi:10.1053/j.gastro.2011.06.057)
Supplement: Supplementary Data [file mmc1.pdf]

SUPPLEMENTARY FIGURE METHODS AND LEGENDS

Methods:

DC stimulation. Purified DC subsets were stimulated overnight with 1μg/ml lipopolysaccharide. Harvested supernatants were analysed for cytokine secretion via cytometric bead array on a FACS Array.

Figures:

Supplementary Figure 1: Alteration of RA function does not rescue the enhanced Treg inducing abilities of CD103+ mLN DCs lacking αvβ8 integrin. CD103+/- DCs isolated from the mLN of control or *Itgb8 (CD11c-Cre)* mice were cultured with CD4+ Foxp3^{GFP}- T-cells plus anti-CD3 antibody without addition of exogenous active TGFβ, in the presence of **(A)** 100nM RA and/or 40μg/ml anti-TGFβ antibody or **(B)** 1μM of RA inhibitors and/or 40μg/ml anti-TGFβ antibody. Cells were analysed by flow cytometry after 5 days for cells that were Foxp3^{GFP}+. Data is representative of 2 independent experiments. N.S. = not significant.

Supplementary Figure 2: Lack of αvβ8 integrin expression by intestinal DCs does not alter their ability to produce active retinoic acid. (A) RNA was purified from control or αvβ8-/- CD103+/- DCs and analysed for *aldh1a2* expression by quantitative RT-PCR. Data shown is representative of 2 independent experiments **(B)** CD103+/- DCs from control or *Itgb8 (CD11c-Cre)* mice mLN were cultured with CD4+ Foxp3^{GFP}- T-cells in the presence of anti-CD3 Ab and the absence of exogenous active TGFβ. Expression of the RA-inducible receptor integrin α4β7 and Treg marker Foxp3 on CD4+ T-cells was analysed by flow cytometry after 5 days. Representative plots are shown from 3 independent experiments, gated on CD4+ cells.

Supplementary Figure 3: Lack of αvβ8 integrin expression by intestinal CD103+ DCs does not result in a pro-inflammatory CD103- DC-like response. Control or integrin αvβ8-/- CD103+/- DCs were incubated for 24hrs in the presence of 1µg/ml L.P.S. for 24hrs. Cytokines were detected in supernatants via cytometric bead array analysis. Data represents means of 3 independent experiments. *P<.05.

Supplementary Figure 4: Lack of αvβ8 integrin expression by gut DCs does not alter the levels of MT1-MMP which are comparable between CD103+/- DC subsets. RNA was purified from control or αvβ8-/- CD103+ and CD103- DCs and analysed for MT1-MMP expression by quantitative RT-PCR. Data from 2 independent experiments.

Supplementary Figure 5: Integrin αvβ8-mediated TGFβ activation is critical for the enhanced ability of CD103+ DCs to induce Foxp3+ Tregs in the presence of CD44^{high} CD4+ T-cells. Foxp3+ Treg induction assays were performed using either control or integrin αvβ8-/- CD103+/- DCs and CD4+ Foxp3^{GFP}- T-cells including the CD44^{high} subset at a CD44^{high} /CD44^{low} ratio of 1:4, in the presence of 40µg/ml mIgG or anti-TGFβ antibody with/without 100nM RA. Data **(A)** and representative flow cytometry plots **(B)** are representative of 2 independent experiments.

Supplementary Table 1: Sequence of primers used to detect gene products by quantitative PCR. Primer pairs (forward and reverse) were used to detect indicated gene products by quantitative PCR as detailed in the Materials and Methods section.

SUPPLEMENTARY FIGURE 1

A.

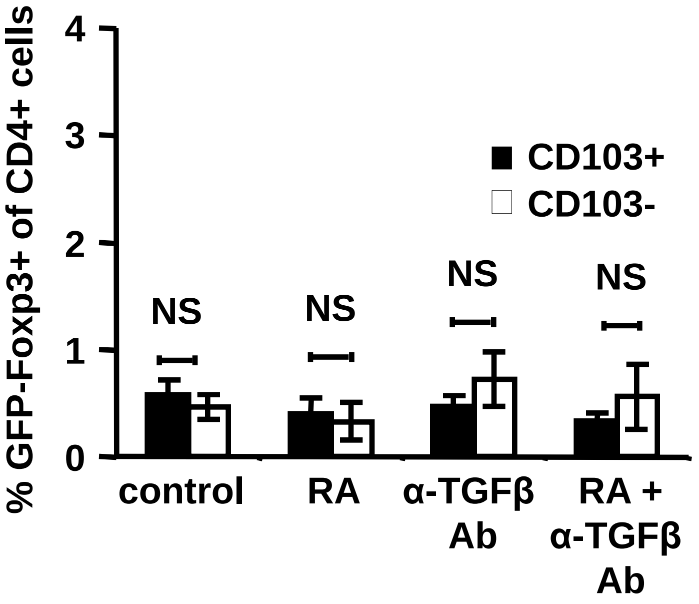

B.

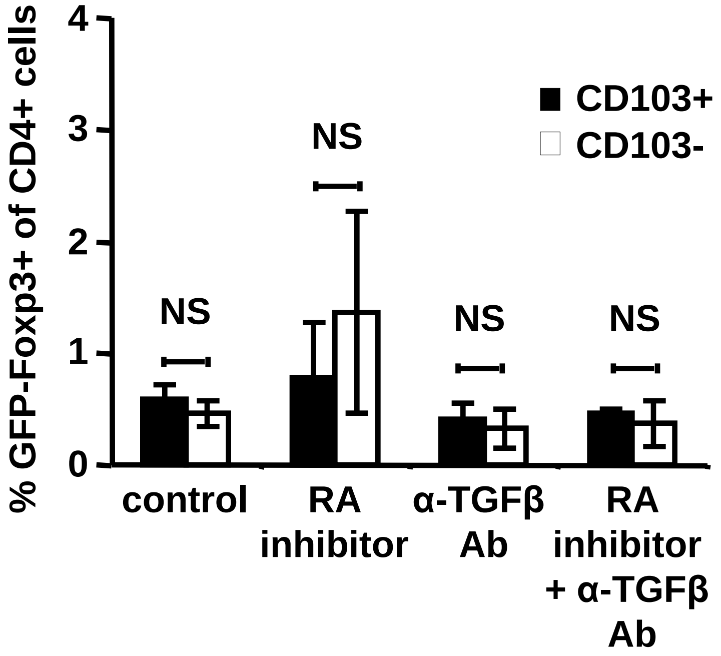

SUPPLEMENTARY FIGURE 2

A.

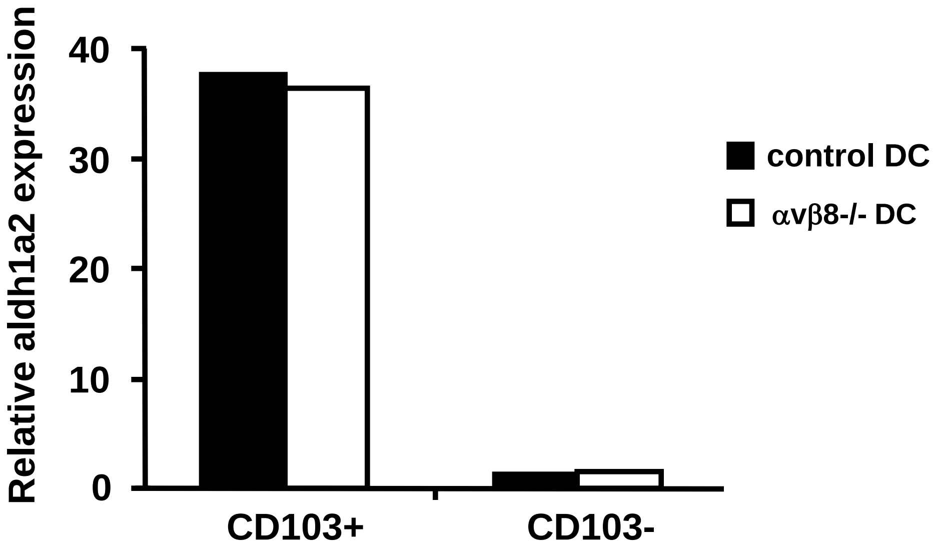

B.

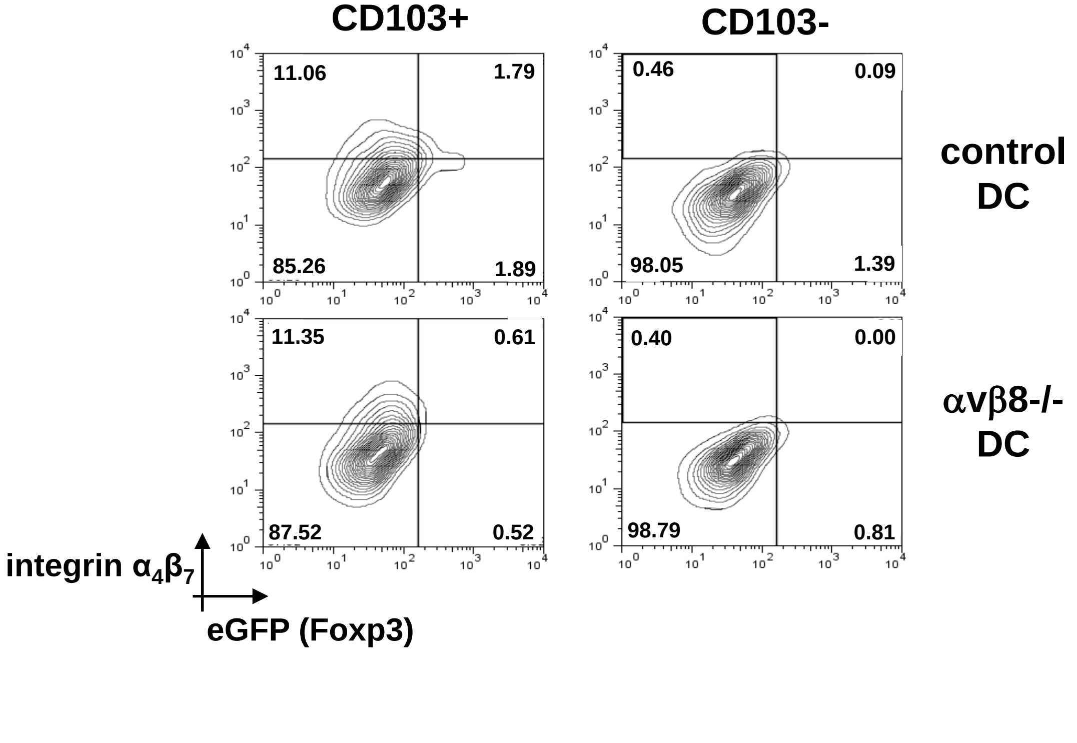

SUPPLEMENTARY FIGURE 3

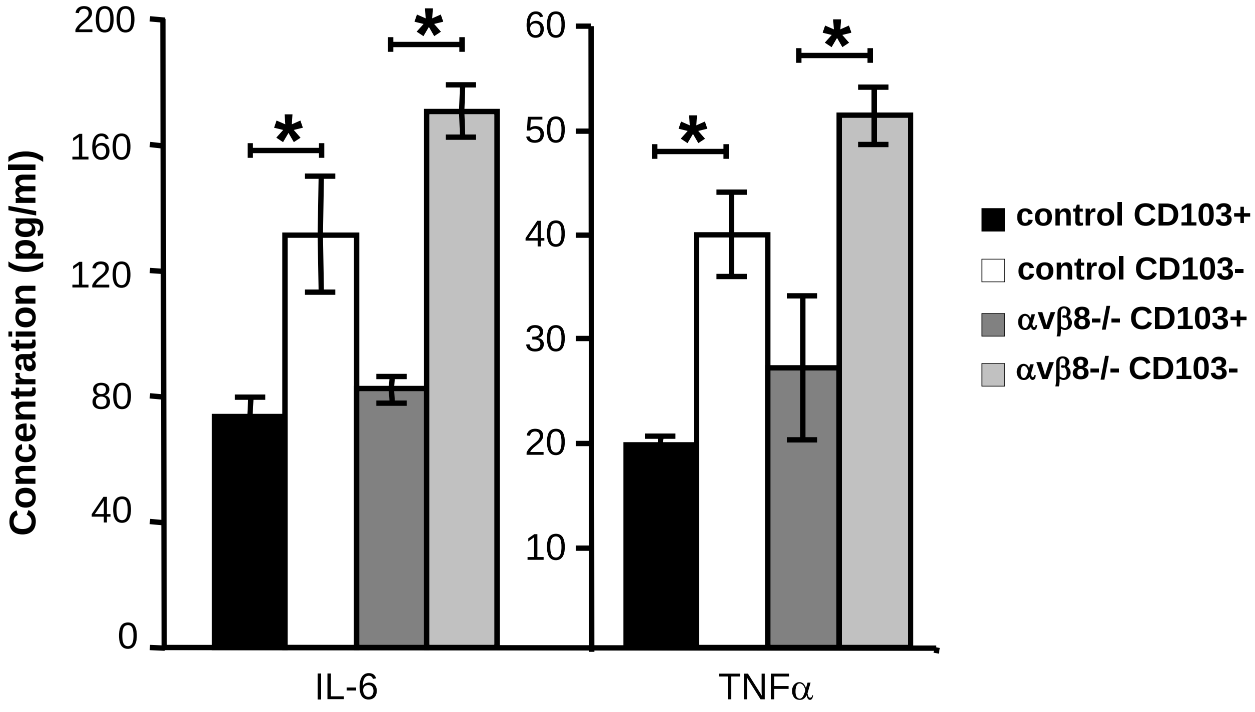

SUPPLEMENTARY FIGURE 4

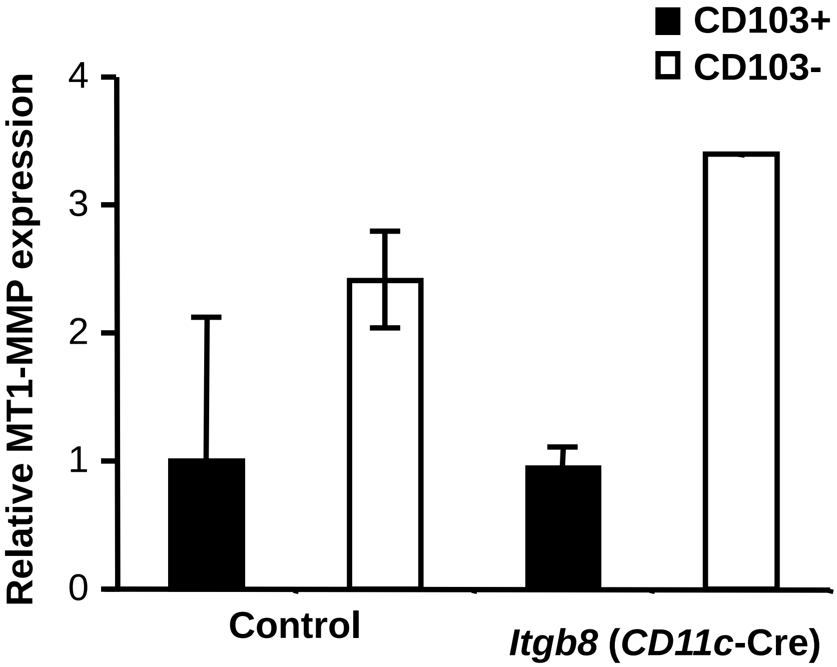

SUPPLEMENTARY FIGURE 5

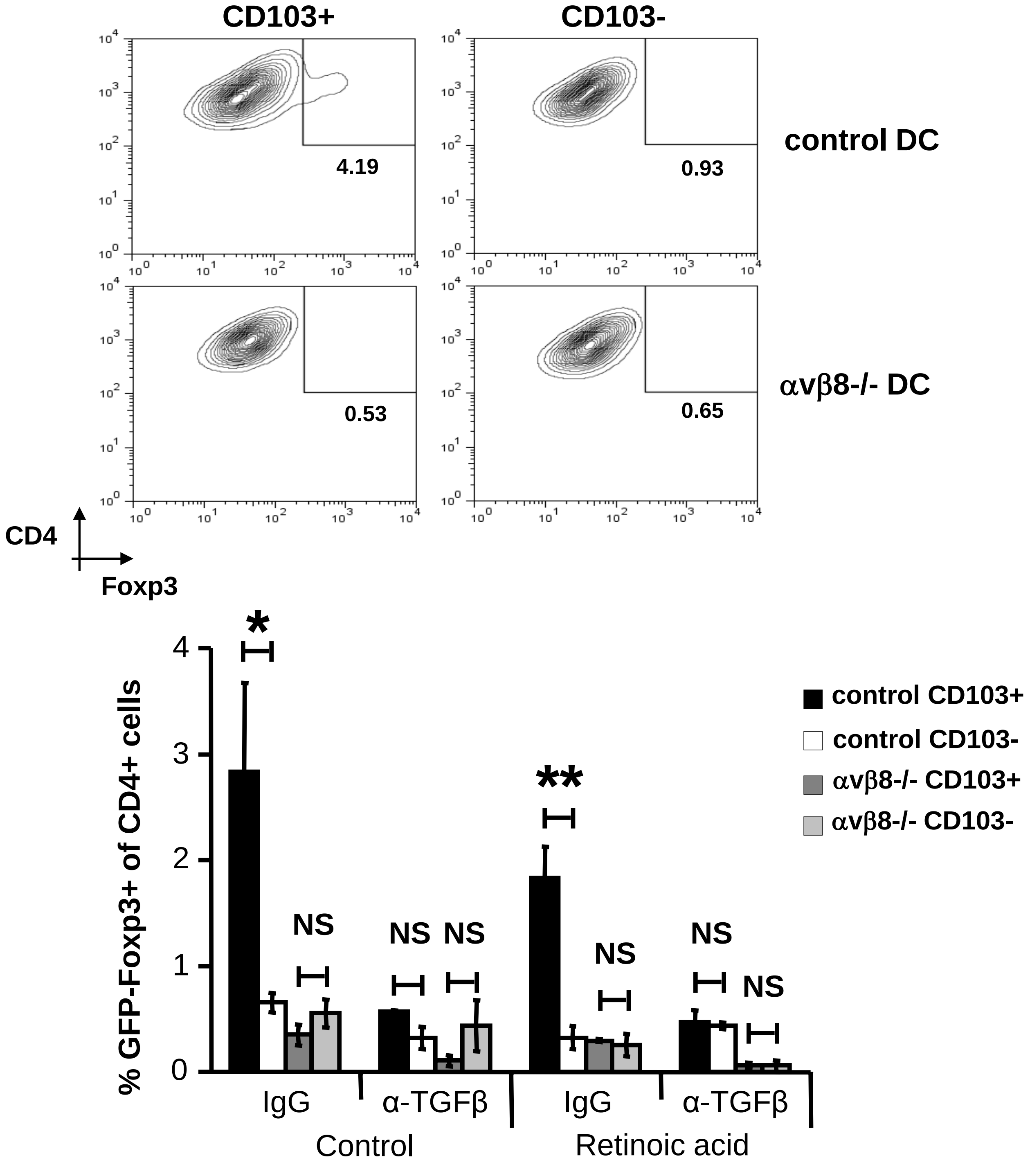

SUPPLEMENTARY TABLE 1

Primer	Sequence
HPRT Forward	GCGTCGTGATTAGCGATGATGAAC
HPRT Reverse	GAGCAAGTCTTTCAGTCCTGTCCA
Integrin β8 Forward	GGGTGTGGAAACGTGACAAGCAAT
Integrin β8 Reverse	TCTGTGGTTCTCACACTGGCAACT
aldh1a2 Forward	TGTAATCCAGCCACAGGAGAGCAA
aldh1a2 Reverse	ACGTCCTCTTTCAGACGCATCCAT
TGFβ1 Forward	TAAAGAGGTCACCCGCGTGCTAAT
TGFβ1 Reverse	ACTGCTTCCCGAATGTCTGACGTA
MT1-MMP Forward	AGATCAAGGCCAATGTTCGGAGGA
MT1-MMP Reverse	AATGTGGCATACTCGCCCACCTTA
